# Acute Gastroenteritis Caused by GI/2 Sapovirus, Taiwan, 2007

**DOI:** 10.3201/eid1407.071531

**Published:** 2008-07

**Authors:** Fang-Tzy Wu, Tomoichiro Oka, Naokazu Takeda, Kazuhiko Katayama, Grant S. Hansman, Chih-Hsin Muo, Shy-Yuan Liang, Ching-Hsiang Hung, Donald Dah-Shyong Jiang, Jui Hsin Chang, Jyh-Yuan Yang, Ho-Sheng Wu, Chen-Fu Yang

**Affiliations:** * Centers for Disease Control, Taipei, Taiwan, Republic of China; † National Institute of Infectious Diseases, Tokyo, Japan

**Keywords:** Sapovirus, gastroenteritis, outbreak, letter

**To the Editor:** Sapovirus is an etiologic agent of human gastroenteritis. Although many of the previously reported cases were of mild, sporadic infections in young children (*1*–*3*), several recent sapovirus-associated gastroenteritis outbreaks have affected adults, which suggests that the virus’s virulence, prevalence, or both, may be increasing (*4*–*6*). In this study, we describe a sapovirus-associated outbreak of gastroenteritis that occurred during May 4–8, 2007, and involved college students in northern Taiwan.

A total of 55 students had clinical symptoms of gastroenteritis, including diarrhea (45), vomiting (22), abdominal cramps (17), and fever (2). The clinical symptoms continued for up to 10 days (mean 4.7 days). Stool specimens were collected from 8 of 55 students on May 8 (Table). Initially, the specimens were screened for bacteria, rotavirus, and norovirus, but all specimens were negative for these pathogens. The 8 stool specimens were then examined by electron microscopy (EM), and 1 was positive for calicivirus-like particles.

**Table:** Clinical symptoms and laboratory diagnosis results for sapovirus-related outbreak among college students, northern Taiwan, May 2007*†

Specimen no.	Patient sex/age, y	Date of illness onset	EM results	RT-PCR results	Copies cDNA/g of stool‡	Symptom			
						Fever	Diarrhea	Vomiting	Abdominal pain
1	F/20	May 5	–	+	1.69 × 10^8^	–	+	+	+
2	F/26	May 5	–	+	6.19 ×10^8^	–	+	+	–
3	M/19	May 6	–	+	2.32 × 10^8^	–	+	–	+
4	M/18	May 6	–	+	3.24 × 10^8^	–	+	+	+
5	F/21	May 7	+	+	1.72 × 10^10^	–	+	–	–
6	F/18	May 4	–	–	–	+	+	+	–
7	M/19	May 7	–	+	4.28 × 10^8^	–	+	+	+
8	F/20	May 6	–	+	2.86 × 10^7^	–	+	–	+

*EM, electron microscopy; RT-PCR, reverse transcription–PCR; –, negative; +, positive. †All specimens were collected May 8. ‡cDNA copies were determined by real-time PCR.

To confirm the EM results, we performed reverse transcription–PCR (RT-PCR), real-time RT-PCR, and sequence analysis as previously described (*7*). Briefly, purified RNA (10 μL) was reverse transcribed by using SuperScript III reverse transcriptase according to the manufacture’s instructions (Invitrogen, Carlsbad, CA, USA). PCR was carried out by using the SV-F11 and SV-R1 primer set directed against the conserved N terminal capsid region (*8*). The PCR products were analyzed with 2% agarose gel electrophoresis and visualized after ethidium bromide staining. The PCR-generated amplicons (≈780 bp) were excised from the gel and purified by the QIAquick gel extraction kit (QIAGEN, Hilden, Germany).

Nucleotide sequences were prepared with the terminator cycle sequence kit (version 3.1) and determined with the ABI 3130 sequencer (Applied Biosystems, Foster City, CA, USA.). Nucleotide sequences were aligned by using ClustalX (www.clustal.org), and the distances were calculated by using the Kimura 2-parameter method. A phylogenetic tree was generated by the neighbor-joining method as described previously (*1*,*8*).

Of the 8 specimens, 7 were positive by RT-PCR and real-time RT-PCR (Table). SaV124F, SaV1F, SaV5F, and SaV1245R primers as well as SaV124TP and SaV5TP minor-groove binding probes were used for real-time RT-PCR diagnosis, which targets the sapovirus RdRp-capsid junction region as described (*7*). The number of sapovirus cDNA copies ranged from 2.86 × 10^7^ to 1.72 × 10^10^ copies/g of stool specimen; mean was 2.71 × 10^9^ copies/g of stool specimen (Table). Sequence analysis of the 7 positive specimens showed 100% nucleotide identity (nt 5098–5878), indicating that the outbreak was caused by 1 sapovirus strain.

To better classify the sapovirus, we reamplified the 3′ end of the genome from 1 positive specimen and sequenced ≈2,400 nt (nt 5074-3′) (Hu/SaV/9–5/Taipei/07/TW; GenBank accession no. EU124657). PCR was performed with SV-F13, SV-F14, and TX30SXN primers as described (*1*). Database searches found a closely matching sapovirus sequence (99%) that was detected in a patient with gastroenteritis in Japan, in 2004 (Chiba041413 strain; GenBank accession no. AB258427). The next closely matching sequence was detected in an outbreak of gastroenteritis among adults in the United States in 1994 (Parkville strain; HCU73124) (*6*). Phylogenetic analysis clustered these 3 sapovirus sequences into genogroup I/genotype 2 (GI/2) (Appendix Figure).

Sapovirus was reported in Japan in water samples (untreated wastewater, treated wastewater, and a river) and in clam samples intended for human consumption (*1*). Apart from these 2 environmental studies, little is known about reservoir of sapovirus or its route of infection in the natural environment. The source of contamination in this current outbreak was not determined; however, none of the food handlers associated with the college reported symptoms of gastroenteritis. However, in a recent molecular epidemiologic study in Japan, a large number of symptomatic and asymptomatic food handlers were found to be infected with noroviruses (*9*). Several seroprevalence studies also indicated high prevalence rates of antibodies to sapovirus in adults and children (*10*). All of these findings highlight the need to collect stool specimens from asymptomatic persons and indicate possible “silent” transmission through an asymptomatic route. Symptoms of sapovirus infection are thought to be milder than symptoms of norovirus infections. However, in this study approximately one third (17) of the 55 students reported symptoms of abdominal pain and 22 (40%) reported symptoms of vomiting. Many of the earlier sapovirus studies described sapovirus GI/1 infections in young Japanese children (*1*), which indicated that infecting virus had a different genotype than the virus detected in this study (GI/2).

In addition, the viral load in this study appeared to be comparatively high. These results suggest that some sapovirus genotypes are more virulent than others. Similar findings were obtained with norovirus infections around the world; strains belonging to norovirus GII/4 were the most prevalent in many countries. Although several recombinant sapovirus strains have been identified and found to be the cause of increased numbers of infections in some countries (*1*,*5*), they were not observed in this study. Increased sapovirus surveillance and reporting are needed to shed some more light on this poorly understood virus.

## Supplementary Material

Appendix Figure Phylogenetic analysis of sapovirus capsid nucleotide sequence showing the close relatedness of Taiwan strain Hu/SaV/9-5/Taipei/07/TW to Chiba041413 (genogroup GI/2). The numbers on each branch indicate the bootstrap values for the genotype. Bootstrap values of 95% or higher were considered statistically significant for the grouping. Scale bar represents nucleotide substitutions per site. GenBank accession numbers for the reference strains are as follows (from top): Parkville, U73124; Houston27, U95644; Potsdam, AF294739; Hu/SaV/9-5/Taipei/07/TW, EU124657; Chiba041413, AB258427; Stockholm318, AF194182; Ehime643, DQ366345; Chiba000496F, AJ412800; Sapporo, U65427; Manchester, X86560; SW278, DQ125333; Ehime1107, DQ058829; Arg39, AY289803; NK24, AY646856; Cruise ship, AY289804; C12, AY603425; Mc10, AY237420; Mex340, AF435812; Mc2, AY237419; PEC, AF182760. Boldface indicates the strain isolated in this study.
