# Supplementary figures and images for: Acute Gastroenteritis Caused by GI/2 Sapovirus, Taiwan, 2007
Source: Emerg Infect Dis. 2008 Jul;14(7):1169–71. doi: 10.3201/eid1407.071531 (PMC2600344; doi:10.3201/eid1407.071531)

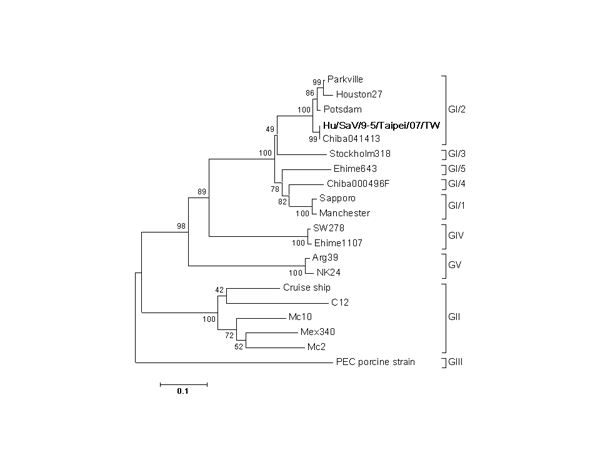

Supplement: Appendix Figure — Phylogenetic analysis of sapovirus capsid nucleotide sequence showing the close relatedness of Taiwan strain Hu/SaV/9-5/Taipei/07/TW to Chiba041413 (genogroup GI/2). The numbers on each branch indicate the bootstrap values for the genotype. Bootstrap values of 95% or higher were considered statistically significant for the grouping. Scale bar represents nucleotide substitutions per site. GenBank accession numbers for the reference strains are as follows (from top): Parkville, U73124; Houston27, U95644; Potsdam, AF294739; Hu/SaV/9-5/Taipei/07/TW, EU124657; Chiba041413, AB258427; Stockholm318, AF194182; Ehime643, DQ366345; Chiba000496F, AJ412800; Sapporo, U65427; Manchester, X86560; SW278, DQ125333; Ehime1107, DQ058829; Arg39, AY289803; NK24, AY646856; Cruise ship, AY289804; C12, AY603425; Mc10, AY237420; Mex340, AF435812; Mc2, AY237419; PEC, AF182760. Boldface indicates the strain isolated in this study. [file 07-1531_app-s1.gif]
